# A Review of Current and Evolving Imaging Techniques in Cardiac Amyloidosis

**DOI:** 10.1007/s11936-023-00976-7

**Published:** 2023-03-04

**Authors:** Rola Khedraki, Austin A. Robinson, Timothy Jordan, Justin L. Grodin, Rajeev C. Mohan

**Affiliations:** 1 Section of Advanced Heart Failure, Division of Cardiovascular Medicine, Scripps Clinic, Prebys Cardiovascular Institute, 9898 Genesee Ave., AMP-300, La Jolla, San Diego, CA 92037, USA; 2 Division of Cardiology, Department of Medicine, University of Texas Southwestern Medical Center, Dallas, USA

**Keywords:** Cardiac amyloidosis, Bone scintigraphy, Echocardiography, Magnetic resonance imaging, Transthyretin, Light-chain amyloidosis

## Abstract

**Purpose of review:**

Establishing an early, efficient diagnosis for cardiac amyloid (CA) is critical to avoiding adverse outcomes. We review current imaging tools that can aid early diagnosis, offer prognostic information, and possibly track treatment response in CA.

**Recent findings:**

There are several current conventional imaging modalities that aid in the diagnosis of CA including electrocardiography, echocardiography, bone scintigraphy, cardiac computed tomography (CT), and cardiac magnetic resonance (CMR) imaging. Advanced imaging techniques including left atrial and right ventricular strain, and CMR T1 and T2 mapping as well as ECV quantification may provide alternative non-invasive means for diagnosis, more granular prognostication, and the ability to track treatment response.

**Summary:**

Leveraging a multimodal imaging toolbox is integral to the early diagnosis of CA; however, it is important to understand the unique role and limitations posed by each modality. Ongoing studies are needed to help identify imaging markers that will lead to an enhanced ability to diagnose, subtype and manage this condition.

## Introduction

**Fig. 1:**
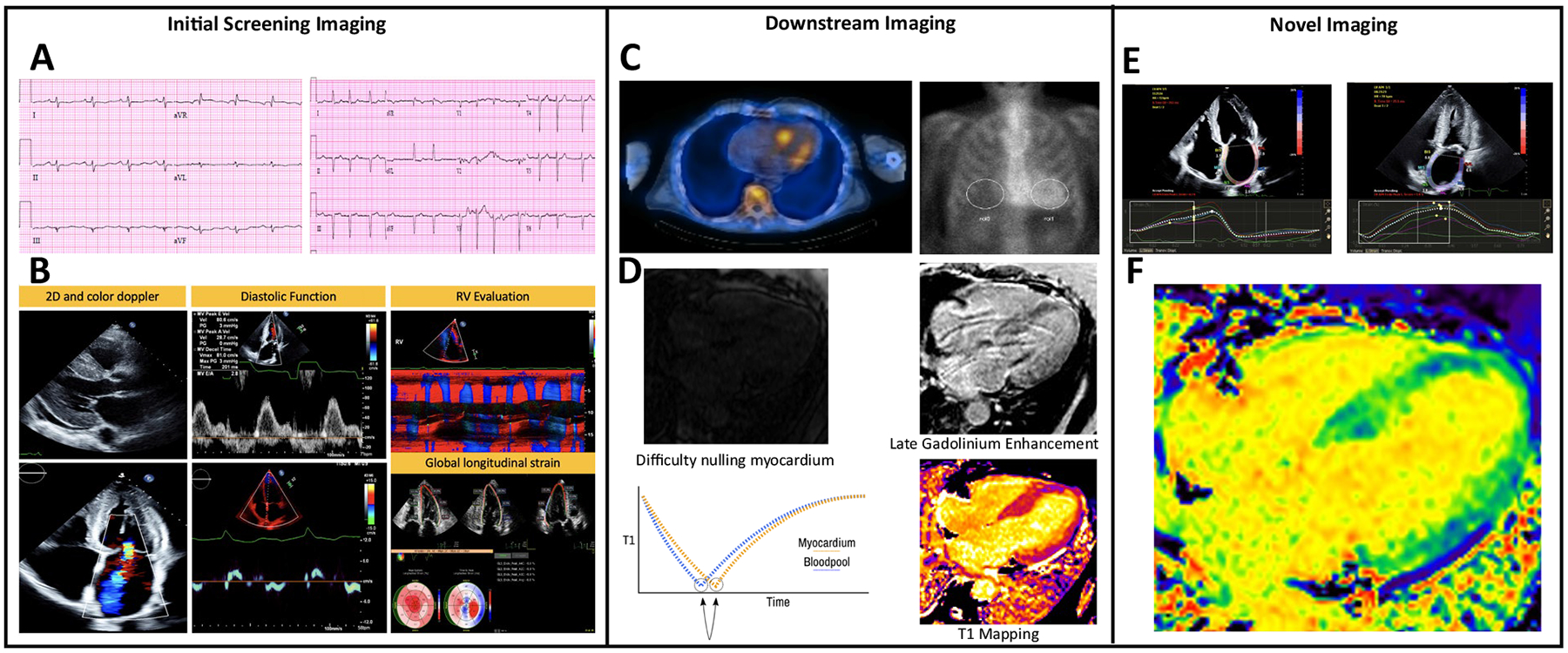
**A** ECG evaluation with low voltage is a commonly cited feature in CA (left panel); however, it is important to be cognizant of other ECG features such as voltage out of proportion to LV mass as well as pseudo-infarct pattern (right panel) and atrial fibrillation which is a common arrhythmia seen (right panel). **B** Echocardiographic assessment commonly shows LV thickening, occasionally with a pericardial effusion (top left), thickened and/or regurgitant valves (bottom left), while mitral inflow evaluation can show a restrictive diastolic pattern (top middle panel) and low tissue Doppler (bottom middle panel). RV evaluation with low TAPSE can suggest significant RV involvement and is a poor prognostic feature (top right). The characteristic global longitudinal strain pattern in CA is apical sparing which is also associated with poorer prognosis (bottom right). **C** Tc99m-PYP scanning showing SPECT/CT image on the left and planar imaging on the right with region of interest (ROI) drawn over the myocardium and ROI over the contralateral side in order to evaluate a heart/contralateral ratio. **D** Cardiac MRI images showing features consistent with CA. **E** LA strain has emerged as another means of evaluating for atrial myopathy as a result of amyloid infiltration and has been shown to have strong correlation with survival **F** ECV measurement by cardiac MRI.

Cardiac amyloidosis (CA) is the result of deranged protein misfolding leading to amyloid fibril deposition into the extracellular space. This inevitably leads to a syndrome of progressive restrictive cardiomyopathy, myocardial conduction abnormalities, and clinical heart failure. The two main types of cardiac amyloidosis are categorized as either transthyretin amyloid (ATTR) or light chain amyloidosis (AL). ATTR CA is further subcategorized as wild type (ATTRwt) or hereditary (hATTR). The advent of disease-modifying therapies, including stabilizer and gene silencer therapies for ATTR and novel chemotherapeutics for AL amyloid have spurred an intense and increased interest in diagnosis. Importantly, prognosis hinges on a timely diagnosis to guide appropriate therapies. In this review, we will present the various non-invasive multimodal imaging tools available including current and novel approaches and techniques which can facilitate earlier diagnosis or show promise in helping to track the progression and response to therapy (Fig. 1, Table 1).

## Brief overview

Clinical suspicion for CA often hinges on a characteristic constellation of clinical signs and symptoms, often coupled with elevations in cardiac biomarkers such as NT pro BNP and/or troponin. A targeted diagnostic approach for CA rests on serum and urine immunofixation and serum-free light chains in conjunction with one or more of the imaging modalities reviewed below [1••]. Electrocardiography (ECG) is one of the most accessible diagnostic tools available for raising suspicion for CA, particularly when combined with a targeted review of systems and echocardiography [2]. The classic features of ECG are low voltage or voltage defined as < 5 mm in the limb leads or < 10 mm in the precordial leads. However, low voltage out of proportion to the degree of left ventricular (LV) mass can be another clue [3••, 4••]. This is attributed to amyloid deposition in the extracellular space which interferes with voltages generated. However, this is a nonspecific criterion and the absence of low voltage does not rule out CA as it is possible for patients with CA to even meet ECG criteria for left ventricular hypertrophy [3••, 4••, 5•]. Nonetheless, when present, low voltage does track with prognosis in CA [6, 7]. Other ECG clues include a pseudo-infarct pattern, conduction abnormalities, and atrial fibrillation [2, 3••, 4••].

## Echocardiography

One of the first essential clues in raising suspicion for amyloidosis is echocardiography, as it is a mainstay in the evaluation of patients with dyspnea or heart failure symptoms. Increased LV wall thickness, particularly when combined with relative low voltage on ECG is a classic manifestation of CA. This is a direct consequence of amyloid infiltration rather than myocyte hypertrophy. Several echocardiographic defining features can often point towards the need for further work up including increased LV and right ventricular (RV) wall thickness, biatrial enlargement, interatrial septal thickening, thickened atrioventricular valves, pericardial effusions, and restrictive LV filling [1••, 8]. Pericardial effusions and valvular insufficiency, although nonspecific findings, are associated with a worse prognosis in CA [9].

### Left ventricle

While echocardiography is not sufficient in distinguishing amyloid subtypes, it has been suggested that LV wall mass and thickness are typically higher in patients with ATTRwt as compared to hATTR or AL likely due to a more insidious disease process with longer exposure of amyloid aggregation and accumulation into the myocardial extracellular space [8, 10]. LV ejection fraction (EF) is often preserved despite a reduction in stroke volume. This is due to a reduction in left ventricular end-diastolic volume (LVEDV), and therefore, the ratio of stroke volume to LVEDV, which represents EF, is often normal. For this reason, EF is thought to poorly capture the pathological changes seen in CA, and it has been proposed that a more meaningful metric is myocardial contraction fraction (MCF), or the ratio of stroke volume to myocardial volume with normal values being 60–65% [11–13]. In fact, a reduced stroke volume despite normal or even “supra-normal” EF is associated with a higher risk of adverse outcome in CA [14–17]. However, systolic dysfunction can occur in more advanced stages of the disease due to cardiomyocyte damage and fibrotic changes over time [8]. Reduced EF at presentation is more common in the hereditary Val122Ile variant than in ATTRwt which commonly affects African American patients and is likely reflective of more advanced disease at diagnosis [18, 19].

### Global longitudinal strain

Myocardial strain, which allows for a quantitative assessment of myocardial deformation, is a useful adjunct in the echocardiographic evaluation for CA. The characteristic LV longitudinal strain pattern in CA is sparing of the apical segment as compared to the impaired basal and mid myocardial segments and is often referred to as a “cherry on top” or “bull’s eye plot.” Although not diagnostic, an apical sparing pattern can be helpful in distinguishing CA from other etiologies such as hypertrophic cardiomyopathy, aortic stenosis, or hypertensive heart disease with a sensitivity of about 88% and specificity of 72% [5•, 20–22]. Importantly, decreased global longitudinal strain is an independent predictor of mortality irrespective of EF [1••, 8, 10, 23, 24]. Lower strain values are typically seen in AL CA as compared to ATTR CA for any given wall thickness which is thought to be secondary to direct light-chain myocardial toxicity and is strongly associated with worse outcomes [8, 10, 23].

### Left atrial myopathy and left atrial strain

Historically, left atrial (LA) function has been inferred from size; however, other metrics for defining LA function are emerging as key in the assessment of overall cardiac performance and thromboembolic risk in various cardiac disorders [25•]. LA strain has important pathophysiologic, diagnostic, and prognostic implications in CA, however, has not been adopted into routine clinical practice outside of non-academic or research settings due to the need for operator input and time, making it impractical particularly in community settings. Nonetheless, LA function as measured by speckle-tracking echocardiography has been shown to be severely reduced in CA patients, both in AL and ATTR [26]. Bandera et al. utilized echo speckle-tracking strain imaging on a cohort of 906 pts with ATTR CA, and showed a substantial impairment of the 3 functions of the LA (reservoir, conduit, contraction) as well as increased atrial stiffness (the resistance to deformation of the LA), with increased LA stiffness correlating with worse prognosis [27••]. Their study also identified 20% of the study population as having loss of atrial contraction despite sinus rhythm (atrial electromechanical dissociation) and noted that this population had significantly poorer prognosis than patients with sinus rhythm who maintained effective mechanical contraction [27••].

Notably, CA patients have an incremental thromboembolic risk independent of atrial fibrillation (AF), with several studies suggesting atrial myopathy as the underlying etiology for this finding [27••, 28–30]. In fact, it has been a suggested practice to prophylactically offer anticoagulation to patients with CA and a diminutive A wave on mitral inflow evaluation or abnormal atrial strain suggestive of atrial mechanical failure [8, 27••]. Intracardiac thrombus has also been documented in patients with sinus rhythm, which might be explained by the previously mentioned concept of atrial electromechanical dissociation [27••, 31•, 32]. Prior studies have also demonstrated a high prevalence of intracardiac thrombus despite anticoagulation, with one study showing a 13.1% prevalence of intracardiac thrombus as detected on cardiac MRI (CMR) in an anticoagulated CA population with AF. Therefore, it is recommended on the basis of clinical experience at high-volume amyloid centers to routinely perform imaging with CMR or transesophageal echo (TEE) prior to attempts at direct current cardioversion regardless of anticoagulation status [30, 33•].

### Right ventricle

The RV has a unique structure and function and differs from the LV with different myocardial fiber arrangement, cavity wall thickness, and structural make-up of the inflow and outflow tracts. A host of echocardiographic parameters, including dimension and function measures, has elucidated the impact of CA on the right heart. In a prior study comparing patients with CA vs. controls, those with CA showed increased RV size, RV basal diameter, IVC, RV wall thickness resulting in reduced total RV volume, and reduced systolic function as measured by tricuspid annular plane systolic excursion (TAPSE) [34]. Another study of AL patients investigated echocardiogram and doppler myocardial imaging (DMI) right heart parameters and found evidence of abnormal RV systolic function by TAPSE and DMI modalities, with a systolic strain of the basal segment of the RV free wall and TAPSE being most useful in distinguishing AL patients from controls [35]. Multiple studies have shown evidence of abnormal change in free wall RV strain correlating with worse prognosis in CA patients [35–38]. Additionally, TAPSE derived by both echocardiography and CMR has shown a strong correlation with worse prognosis in CA patients [15, 34, 39].

## Bone scintigraphy

Contemporary diagnostic algorithms incorporate bone scintigraphy as part of the evaluation for CA, but only after laboratory screening evaluation to rule out AL amyloidosis using serum and urine immunofixation and serum-free light chains [40, 41••]. The most common radiotracer used in the USA for this purpose is technetium-99 m (Tc99m) which is bound to pyrophosphate (PYP). In the USA, PYP is the only available agent while other agents such as 3,3-diphosphono1,2-propanodicarboxylic acid are available in other parts of the world. The exact mechanism of uptake of tracer into the myocardium in the setting of CA is not fully understood but thought to be related to calcium deposition [42, 43••].

The rise in the use of bone scintigraphy as part of the evaluation for CA stems from the results of a large, multicenter study which evaluated patients with suspected CA. An abnormal bone scintigraphy study showing myocardial uptake in conjunction with a negative evaluation for a monoclonal gammopathy provided a specificity and positive predictive value of 100% for ATTR —paving the way for a nonbiopsy diagnosis of cardiac amyloidosis [1••]. Due to the diagnostic accuracy in identifying ATTR CA, this has led to less reliance on endomyocardial biopsy for confirmatory diagnosis, thus minimizing the potential for procedural-related risk to the patient. Furthermore, in a large analysis of Medicare beneficiaries, the incidence and prevalence of CA increased significantly between 2000 and 2012, particularly after 2006 [44, 45]. Therefore, bone scintigraphy has not only been practice changing in reducing the need for biopsy, but has also led to increased incidence of disease which has coincided with an increase in the availability of noninvasive imaging techniques to evaluate for CA, as endomyocardial biopsy is not widely available at all centers. However, biopsy continues to play an important role as the gold standard in settings in which there is discordant data or equivocal serologic or imaging workup.

Bone scintigraphy is performed by obtaining both planar and single photon emission computed tomography (SPECT) images after injection of the Tc99m-PYP tracer. The images are then assessed for the presence of a tracer in the myocardium. A quantitative evaluation based on planar images can be done in which the total counts are tabulated in a region of interest (ROI) over the heart and compared to a ROI over the contralateral lung. A ratio of ≥ 1.5 is considered positive for the presence of ATTR-CA using a 1-h protocol or ≥ 1.3 using a 3-h protocol [46••]. Additionally, a semiquantitative assessment is performed by comparing the uptake of the tracer in the heart as compared to nearby rib uptake. Myocardial uptake equal to (grade 2) or greater than rib uptake (grade 3) is considered suggestive of ATTR-CA [46••]. However, a key feature of interpretation relies on SPECT imaging for confirmation as described below.

A common pitfall in the interpretation of these tests is the presence of a tracer in the blood pool, or left ventricular cavity as opposed to the myocardium itself which may falsely elevate the counts in the ROI over the heart resulting in either an equivocal study or a false positive study when only planar imaging is used. Protocols include both a 1-h delay and a 3-h delay after injection. While a 1-h protocol can increase the throughput of patients, the 3-h protocol may help decrease the possibility of pooling of the tracer in the left ventricular cavity, known as blood pooling. Consequently, planar imaging alone is not enough to confirm the diagnosis of ATTR-CA, and therefore, SPECT is a key component for confirming myocardial uptake [47, 48•, 49•]. Several etiologies for potential false positive and false negative interpretation are reviewed in Table 2.

Radionuclide tracer uptake can vary by myocardial region and intensity. A recent study sought to determine the clinical impact of the intensity of tracer uptake. Despite the highly predictive nature of an abnormal scintigraphy scan, the degree of tracer uptake, as measured by the heart-to-contralateral lung ratio, did not impact clinical outcomes [50•]. However, while the precise mechanism of cardiac uptake is uncertain, more diffuse left ventricular tracer uptake may be associated with a worse prognosis [51••].

Once the diagnosis of CA is made typically patients are followed serially clinically, as there is no consensus on the use of serial bone scintigraphy imaging to assess disease progression. A small study involving 20 patients with ATTR-CA found no difference in the Tc99m-PYP scan at baseline and 1.5 years later [52]. In the clinical setting, therefore, these scans are not typically repeated once the diagnosis is made as their key role remains for the diagnosis of ATTR.

While the use of Tc99m-PYP scintigraphy has significantly facilitated the diagnosis of ATTR CA, evaluation for AL still requires tissue biopsy for evaluation. As a result, there is increased interest in noninvasive imaging techniques, specifically positron emission tomography (PET) and tracers that may help differentiate AL from ATTR. F18-labeled tracers have emerged as a possible answer to this problem. One such tracer, F18-florbetapir was shown to have increased uptake in patients with AL-CA as opposed to ATTR-CA [53]. The promise of these PET tracers is that they bind directly to the amyloid protein and may potentially provide a more sensitive way to detect early disease. Notably, serum amyloid P (SAP) scinitgraphy has been available in Europe to determine whole-body amyloid quantification, however, is not approved in the USA [54, 55]. However, there are other nuclear imaging radiotracers on the horizon including AT-01 (Attralus Inc., San Francisco, California), an amyloid-specific radiotracer that is capable of imaging all types of systemic amyloidosis through PET/CT imaging; however, published clinical trials supporting clinical utility and sensitivity are not yet available.

## Cardiac magnetic resonance

Cardiovascular magnetic resonance (CMR) imaging is the gold-standard modality for assessing structure and function in all four chambers of the heart, but its key strength lies in myocardial tissue characterization [56]. Late gadolinium enhancement (LGE), typically used to identify replacement fibrosis in entities such as myocardial infarction or hypertrophic cardiomyopathy, has long been noted as a feature of cardiac amyloidosis as well [57]. A notable distinction is that in CA, LGE correlates not with replacement fibrosis, but with interstitial amyloid accumulation [58]. The typical LGE pattern of CA is global, predominantly subendocardial LGE. A closely associated feature, considered pathognomonic for CA, is the inability to null the myocardium on the inversion (TI) scout sequence performed in preparation for LGE imaging. In such scenarios, the myocardial extracellular matrix, expanded with amyloid fibrils, retains a similar amount of gadolinium as the blood pool, leading to indistinguishable T1 recovery curves. This, in turn, leads to difficulty in identifying the T1 null point of healthy myocardium, required for LGE techniques.

### T1 mapping

Parametric mapping has allowed for additional CMR characterization of cardiac amyloid. Myocardial T1, which represents interstitial expansion that may be commonly related to myocardial edema or fibrosis, is also lengthened by the matrix expansion of myocardial amyloid deposition. The magnitude of T1 elevation is particularly large in CA, allowing for its distinction from other cardiomyopathies [59]. Importantly, so-called native T1 mapping provides a CMR mechanism to CA evaluation that does not require gadolinium contrast. A key consideration is the dependence of T1 values on magnetic field strength and individual sequence parameters, limiting the ability to standardize diagnostic thresholds [60••].

T1 mapping before and after gadolinium administration allows for quantification of the myocardial extracellular volume fraction (ECV), if the hematocrit is known, which tracks closely with collagen fraction on pathology in non-amyloidosis hearts [61•]. A meta-analysis, comparing the diagnostic and prognostic utility of ECV, found that it had a higher diagnostic odds ratio and hazard ratio for adverse events than LGE and native T1 mapping [62••]. ECV mapping has become an important tool in amyloid evaluation, as it may allow for the identification of early-stage cardiac amyloidosis that precedes the development of LGE [63•]. ECV is strongly prognostic in CA and may allow for tracking disease progression or response to CA therapies [32].

### T2 mapping

T2 mapping also aids in the identification and prognostication of cardiac amyloidosis. T2 is sensitive to myocardial water content and state, and the edema associated with the myocyte toxicity of amyloid proteins leads to elevations in T2 times [64]. Myocardial T2 times appear to be prognostic and track response to treatment in AL, but not ATTR amyloid which is thought to be due to the direct cardiotoxicity of light chains themselves causing a greater degree of edema [65].

The stronger association between T2 and AL, compared with ATTR, has led to a recently proposed scoring system to distinguish AL from ATTR subtypes [66•]. The increased T2 with AL is also remarkable because most other markers of disease severity (LV mass, wall thickness, LGE transmural extent, RV involvement) are associated with ATTR amyloid [67]. Efforts to systematize the use of CMR criteria amyloid sub-typing are ongoing, but contemporary society recommendations do not rely on CMR for distinguishing TTR from AL [68•].

### Diffusion tensor MRI

Another non-contrast magnetic resonance technique that has emerged for the characterization of CA is diffusion tensor MRI (DT-MRI). By tracking water diffusion through tissue, DT-MRI allows the evaluation of myocardial micro-structure, quantifying fractional anisotropy (FA), mean diffusivity (MD), and myocardial sheetlet orientation. Early studies have demonstrated that MD tracks well with ECV estimates across amyloid subtypes, but that diastolic sheetlet changes may differ between ATTR and AL [69]. Additional validation studies and standardization of clinical protocols are the next important steps for this promising technique.

## Cardiac computed tomography (CT)

It has become increasingly recognized that ATTR CA is common in patients with aortic stenosis (AS), particularly in the low-flow low-gradient aortic stenosis phenotype (LFLG AS) [70–72, 73•, 74]. Given the high prevalence of AS in an aging population and the increased use of transcatheter aortic valve replacement (TAVR) therapy in symptomatic patients, pre-procedural cardiac CT has emerged as a potential opportunistic screening tool for ATTR. Prior studies have evaluated patients with severe AS with the addition of ECV quantification to TAVR-planning CT and demonstrated significantly higher ECV values in patients with concomitant ATTR as confirmed by Tc99m-PYP scintigraphy. However, the ECV cutoff threshold needs further validation with larger-scale studies and validation across vendors as well as proposed algorithms for integrating into the current TAVR CT workflow. Importantly, identification of ATTR as a comorbid condition is crucial, as otherwise undiagnosed CA, may attenuate the long-term survival benefit of TAVR.

## Imaging targets in treatment

The advent of rapidly expanding therapeutic options has revolutionized the landscape of CA and has led to increased interest in diagnosis [75, 76, 77••]. In the ATTR space, TTR stabilizers such as tafamidis (Vyndamax/Vydaqel, Pfizer) and TTR gene silencers such as patisiran (Onpattro, Alnylam Pharmaceuticals) and inotersen (Tegsedi, Akcea Therapeutics) are the two main disease-modifying therapies available. However, only tafamidis is approved for the treatment of CA while the silencers are approved for ATTR polyneuropathy with or without cardiac involvement [78, 79]. Treatments for AL, on the other hand, are targeted towards reduction in plasma cell and light chain burden with daratumumab becoming the first-line treatment in the current era [80].

The monitoring of response to therapy continues to be an area of intense research. Although novel treatment strategies have been shown to be clinically effective in reducing circulating biomarkers and improving functional status and symptoms, the translation to concrete imaging markers to tailor therapy is still lacking [81••]. Nonetheless, several studies have attempted to characterize the response to therapy via various imaging modalities, but are generally limited by small patient cohorts.

Echocardiography has been evaluated as a treatment-response monitoring strategy, although with mixed results. In a study evaluating 8 patients with hATTR and 7 patients with ATTRwt, stabilization of echocardiographic parameters including LV wall thickness, LV wall mass, and global longitudinal strain was also demonstrated in patients treated with inotersen [82, 83••]. A sub-study of the APOLLO trial evaluating a subpopulation of patients with CA involvement treated with patisiran showed improvements in echocardiographic markers such as LV wall thickness, LV end-diastolic volume, GLS, and cardiac output compared to placebo at 18 months [84]; however, in patients treated with inotersen, these echocardiographic variables did not differ between treatment and placebo after 15 months of treatment [79, 81••]. In AL disease, although some studies have shown improvement in strain ratio in chemotherapy responders as compared to nonresponders as well as LV size and stroke volume, other studies have failed to show a significant change in these parameters [81••, 85–88].

CMR has also been implemented for treatment monitoring in patients with different CA subtypes and various therapeutics. In a recent prospective study, 176 patients with AL CA were monitored with T1 and ECV mapping at diagnosis and serially at 6, 12, and 24 months after initiation of chemotherapy [89••]. The results of the study were promising and showed that ECV may possibly be used to track decreasing the myocardial amyloid burden and that change in ECV was an independent predictor of prognosis [89••]. Serial native T1 and ECV evaluation using CMR has also been used to monitor for stabilization of disease in ATTR CA patients treated with tafamidis over 12 months; however, large-scale prospective trials are still needed to support this in routine clinical practice [83••, 90]. With regard to gene silencer therapies, a prior study demonstrated a reduction in ECV in 16 patients treated with patisiran when retrospectively matched based on CMR results (adjusted mean difference between groups − 6.2% (95% CI: − 9.5 to − 3.0%); *p* = 0.001) [83••, 91••]. Furthermore, a study evaluating 33 ATTR patients treated with inotersen demonstrated mean LV mass reduction by 8.4% on CMR after 2 years of treatment and reduction by 11.4% at 3 years [83••, 92•].

Although tracking response to therapy is still evolving and current data has shown inconsistent results with regard to serial imaging, CMR-based quantification methods are promising candidates to be at the forefront of this progress given the elegant ability of magnetic resonance to provide high-resolution myocardial tissue characterization. However, the major limitation is cost and standardized protocols. Artificial intelligence (AI)-empowered imaging modalities are also emerging and may prove to be a powerful integration to better stratify patients both for diagnostic purposes and in monitoring response to therapy [83••].

## Conclusions

Despite advances in treatment, CA remains an underdiagnosed and underappreciated condition. With ongoing advances in non-invasive imaging techniques, this diagnostic inertia may be overcome. Accurate diagnosis does not rest on any single technique but the reliance on a multimodal toolbox of integrated and targeted testing. The comprehensive use of current and emerging imaging techniques can help to augment risk stratification, monitoring response to therapy, and subsequent tailored therapy.

## Figures and Tables

**Table 1.** Strengths and limitations of each imaging modality, prognostic features, and emerging techniques for diagnosis and assessing response to treatment

Imaging modality	Strengths	Limitations	Poor prognostic features	Emerging diagnostic techniques/tracking treatment response
Electrocardiogram	Widely accessible Often one of the earliest “red flag” clinical clues to the diagnosis of CA	Nonspecific Low voltage, in particular, can be seen in a variety of conditions including obesity, pericardial effusion, emphysema, pneumothorax, myocarditis The absence of low voltage should not falsely dissuade from the diagnosis of CA [93] Psuedo-infarct pattern may lead to misdiagnosis of coronary artery disease	Fragmented QRS morphology [94] Pseudoinfarct pattern [95] QTc ≥ 483 ms [96] Low voltage as expressed by Sokolow-Lyon index ≤ 1.5 mV (sum of the S wave amplitude in lead V1 and the R wave in V5 or V6 ≤ 15 mm (1.5 mV)) [7]	AI-enabled ECG algorithms have been proposed to aid in diagnosis however larger-scale clinical validation is still needed [97, 98]
Echocardiogram	Widely accessible Important in raising diagnostic suspicion, particularly when combined with ECG features that are concerning for CA GLS showing apical sparing can distinguish CA from other causes of myocardial thickening [22]	Nonspecific Cannot distinguish between AL and ATTR CA The absence of an apical sparing pattern on GLS should not falsely dissuade from the diagnosis of CA [20]	Increased LV mass Restrictive diastolic pattern [99] Pericardial effusion TAPSE < 14 Stroke Volume index < 33 ml/min [16] Myocardial contraction fraction (ratio of LV stroke volume and myocardial volume) < 34% [16, 100] GLS with apical sparing pattern [23, 101]	Left atrial strain has strong association with survival [102, 103], particularly when combined with GLS and RV free wall strain [104], however not currently used in routine clinical practice. Further research is needed to determine whether strain data can be used to guide treatment and provide guidance on generalizability given that strain values are vendor-dependent. Cardiac shear wave (SW) imaging as a novel technique to assess myocardial mechanics with end-diastole SW velocities being higher in CA, and thus, can aid in the diagnostic evaluation [105]
Bone Scintigraphy	High degree of specificity for ATTR which has replaced endomyocardial biopsy when AL is ruled out with laboratory testing	Exposure to ionizing radiation Performance characteristics are dependent on pre-test probability of disease and ruling out a monoclonal gammopathy Multiple pitfalls for false positives (when SPECT not used) and false negatives	Diffuse myocardial uptake [51••] Heart to contralateral ratio > 1.6 [106]	Has been proposed to guide treatment initiation in mutation positive relatives who have not yet manifested ECG or echocardiographic phenotype but have evidence of myocardial uptake on scintigraphy however there is lack of consensus on this approach [107, 108] Currently, no data to support the use of serial imaging with bone scintigraphy to assess treatment response or progression of disease [46••, 109] Positron emission tomography (PET) tracers such as 18F-florbetapir are currently being studied for use in CA and offer advantages of being quantitative rather than qualitative (SPECT) and thus have potential to track amyloid burden and are the only radiotracers that can identify AL amyloid [109]
Cardiac CT	Potential to be used as opportunistic screening method for identification of CA in patients undergoing TAVR CT	Contrast required Exposure to ionizing radiation	No specific CT prognostic criteria, however lowflow, low-gradient AS phenotype is associated with worse outcomes [74]	ECV quantification can be added to TAVR-planning CT to identify patients with severe AS and concomitant CA; however, studies have yet to demonstrate a standardized cutoff and validation across vendors is still warranted, as well as proposed algorithms for integrating into current workflow [110••, 111]
CMR	High image quality with ability to characterize interstitial space Native T1 mapping and diffusion tensor MRI (DT-MRI) are novel techniques that do not require contrast administration	Cannot distinguish between AL and ATTR CA Adding sequences prolongs the duration of the exam Significant postprocessing required Gadolinium is contraindicated in patients with advanced renal disease T1 mapping can be confounded by myocardial edema and platform-dependent variation that can affect normal thresholds [93]	RV involvement [112] Transmural involvement of LGE [63•] Suboptimal ability to null the myocardium [113] Increased ECV Increased T2 times with AL is prognostic however still not standardized for routine clinical use	Left ventricular wall thickness, mass, and ECV are likely useful tools to aid in tracking treatment response [46••] Native T1 and ECV are emerging and are potentially more robust tools than LGE in early disease [114, 115]. ECV has been shown to be effective in assessing regression of AL amyloid with successful chemotherapy and is an independent prognostic marker [89••]. Furthermore, T1 values are higher in AL and ECV values are higher in ATTR, providing potential for discrimination between the two [67, 93] T2 mapping emerging to help distinguish ATTR from AL, given stronger association with AL and could potentially be used to track treatment response in AL [65, 66•] DT-MRI can help to quantify mean diffusivity and myocardial sheetlet orientation and diastolic sheetlet changes may help distinguish ATTR and AL [69]

**Table 2.** Potential etiologies for false positive and false negative interpretation of bone scintigraphy

False positive planar	False positive SPECT	False negative planar/SPECT
Blood pool	Hydroxycholorquine toxicity	Short acquisition time
Rib fracture	Non-ATTR cardiac amyloidosis	Pericardial effusion
Recent myocardial infarction (< 4 weeks)		Early disease
Non-ATTR cardiac amyloidosis		
